# Vitamin D and oral disease relationships: Insights from a bidirectional Mendelian randomization investigation

**DOI:** 10.1097/MD.0000000000046097

**Published:** 2025-11-21

**Authors:** Yuanxin Shi, Xie Li, Bin Chen, Yueyue Wang, Guohui Bai

**Affiliations:** a Key Laboratory of Oral Disease Research, School of Stomatology, Zunyi Medical University, Zunyi, China; b Stomatological Hospital Affliated to Zunyi Medical University, Zunyi, China.

**Keywords:** causal relationships, mendelian randomization, oral diseases, serum 25-hydroxyvitamin D levels

## Abstract

The causal relationship between serum 25-hydroxyvitamin D levels and oral diseases remains uncertain due to confounding and reverse causation in observational studies. This bidirectional 2-sample Mendelian randomization (MR) study aims to elucidate the causal relationship between serum 25-hydroxyvitamin D levels and multiple oral pathologies while addressing confounding factors and reverse causation observed in conventional observational studies. Utilizing summary-level genome-wide association study data from the IEU OpenGWAS project, we analyzed serum 25-hydroxyvitamin D levels (“ebi-a-GCST90000618,” n = 4,96,946 Europeans) and oral disease phenotypes from Finnish biobank registries. Instrumental variables for serum 25-hydroxyvitamin D levels were selected under genome-wide significance (*P* < 5 × 10^−8^) with strict clumping thresholds (*r*^2^ < 0.001, kb = 10,000) and *F*-statistics > 30. For oral disease exposures, relaxed criteria (*P* < 1 × 10^−5^, *r*^2^ < 0.001, *F*-statistic > 10) were applied to ensure sufficient instrumental variables. Primary analyses employed inverse-variance weighted and MR-Egger methods, supplemented by sensitivity analyses including Cochran’s *Q* test, leave-one-out validation, funnel plots, and MR-PRESSO for horizontal pleiotropy assessment. Forward MR analysis demonstrated significant inverse causal relationships between genetically elevated serum 25-hydroxyvitamin D levels and risks of perioral dermatitis and lip, oral cavity, pharyngeal malignancies. No statistically significant associations were observed with other oral diseases. Reverse MR analysis revealed a nominal positive association between lichen planus and serum 25-hydroxyvitamin D levels that did not persist after multiple testing correction, with no other reverse causal effects detected. Our findings provide genetic evidence supporting a protective role of serum 25-hydroxyvitamin D levels against specific oral disorders, particularly perioral dermatitis and oropharyngeal malignancies. The bidirectional design effectively mitigated reverse causation concerns, underscoring the robustness of these causal inferences. These results warrant further investigation into vitamin D supplementation as a potential preventive strategy for targeted oral pathologies.

## 1. Introduction

Oral diseases impose a substantial burden on individuals and society, impairing quality of life and generating significant economic costs.^[1]^ Among various oral pathologies, periodontitis, malignancies of the lip, oral cavity, pharynx, and perioral dermatitis warrant particular attention.

Emerging evidence highlights the potential role of vitamin D a critical regulator of calcium metabolism and immune function in oral health.^[2]^ A systematic review and meta-analysis revealed significantly lower serum vitamin D levels in periodontitis patients compared with healthy controls.^[3]^ Epidemiological studies further demonstrate that vitamin D receptor deficiency correlates with increased incidence of nonmelanoma skin cancers,^[4]^ while cancer patients frequently exhibit severe vitamin D deficiency.^[5]^ Beyond calcium-phosphate homeostasis, the active metabolite 25-hydroxyvitamin D (25(OH)D) appears to influence carcinogenesis through multiple mechanisms, including cell cycle regulation (G0/G1 phase arrest), apoptosis induction via BCL-2 suppression,^[6]^ and immunomodulation of tumor-associated macrophages. Clinical trials suggest vitamin D supplementation may reduce atopic dermatitis severity in pediatric and adult populations,^[7]^ though large-scale longitudinal studies are needed for validation.

Notably, these observational associations remain confounded by lifestyle factors (e.g., UV exposure patterns, dietary habits) and reverse causation – particularly the temporal ambiguity between vitamin D deficiency onset and disease-related behavioral modifications (e.g., sun avoidance in dermatologic malignancies). Conventional observational methodologies cannot differentiate whether hypovitaminosis D directly promotes pathogenesis or merely represents an epiphenomenon of illness-associated behaviors.

Mendelian randomization (MR) leverages naturally occurring genetic variants as instrumental variables (IVs), establishing a framework analogous to randomized controlled trials. This approach significantly enhances the capacity to elucidate causal relationships between exposures and outcomes with minimized confounding bias.^[8,9]^ However, existing MR studies have certain limitations, such as lacking of up-to-date data set analysis^[10]^ and the need for more comprehensive analysis of multiple oral diseases and a deeper understanding of the underlying mechanisms. Therefore, this study aims to fill these gaps by elucidating the causal relationship between serum 25-hydroxyvitamin D levels and multiple oral diseases.

## 2. Materials and methods

### 2.1. Data availability

The IEU OpenGWAS database (https://gwas.mrcieu.ac.uk/) was used to obtain data on serum 25(OH)D levels and various oral diseases. Specific datasets, populations, and sample sizes are shown in Table 1.

**Table 1:** Information on the database.

GWAS ID	Trait	Sample size	Number of SNPs	Population
finn-b-L12_PERIORALDERM	Perioral dermatitis	2,11,324	1,63,80,451	European
finn-b-C3_LIP_ORAL_PHARYNX	Malignant neoplasm of lip, oral cavity and pharynx	2,18,792	1,63,80,466	European
finn-b-CD2_BENIGN_MOUTH_PHARYNX	Benign neoplasm of mouth and pharynx	2,18,792	1,63,80,466	European
finn-b-K11_PERIODON_ACUTE	Acute periodontitis	1,95,762	1,63,80,371	European
finn-b-K11_PERIODON_CHRON	Chronic periodontitis	1,98,441	1,63,80,178	European
finn-b-L12_LICHENPLANUS	Lichen planus	2,14,107	1,63,80,458	European
finn-b-G6_TRINEU	Trigeminal neuralgia	1,95,847	1.63,80,408	European
finn-b-K11_ORAL	Diseases of oral cavity, salivary glands and jaws	2,18,792	1,63,80,466	European
finn-b-K11_ORALCYST	Cysts of oral region, not elsewhere classified	1,95,999	1,63,80,370	European
finn-b-Q17_CLEFT_LIP_CLEFT_PALATE	Cleft lip and cleft palate	2,18,792	1,63,80,466	European
ebi-a-GCST90000618	Serum 25-hydroxyvitamin D levels	4,96,946	68,96,093	European

GWAS = genome-wide association study, SNP = single-nucleotide polymorphism.

All genetic association data were sourced from studies compliant with the Declaration of Helsinki. This MR analysis of summary-level data did not involve direct human/animal experimentation.

### 2.2. Instrumental variable selection

**Figure 1.**
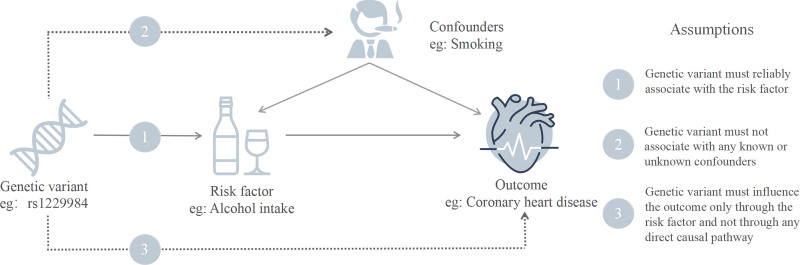
The 3 main assumptions of the MR analysis. MR = Mendelian randomization.

MR analysis relies on 3 core assumptions – relevance: strong association between IVs and the exposure (*P* < 5 × 10^−8^ for forward analysis); independence: IVs are independent of confounders (e.g., smoking); exclusion restriction: IVs influence outcomes solely through the exposure (Fig. 1).

For forward MR (serum 25-hydroxyvitamin D levels to oral diseases), single-nucleotide polymorphism (SNPs) were selected at genome-wide significance (*P* < 5 × 10^−8^) using the twoSampleMR R package. Linkage disequilibrium clumping (*r*² < 0.001, clump distance = 10,000 kb) ensured independence, and SNPs with weak instrument bias (*F*-statistic = β²/SE² < 30) were excluded. The *F*-statistics for all IVs used in the forward MR analyses are provided in Table S1, Supplemental Digital Content, https://links.lww.com/MD/Q701. For reverse MR (oral diseases → serum 25-hydroxyvitamin D levels), relaxed thresholds (*P* < 5 × 10^−6^, *F*-statistic > 10) were applied. Finally, potential pleiotropic SNPs linked to confounders were removed via linkage disequilibrium trait.

Following covariate adjustment procedures, confounding factors associated with lip/oral cavity/pharyngeal malignancies as outcomes were systematically excluded (Table S2, Supplemental Digital Content, https://links.lww.com/MD/Q701). For other investigated diseases and serum 25-hydroxyvitamin D levels treated as outcomes, no significant confounders requiring exclusion were identified through our analytical framework.

### 2.3. MR and sensitivity analyses

This study was conducted and reported in accordance with the Strengthening the Reporting of Observational Studies in Epidemiology using Mendelian Randomization (STROBE-MR) guideline to ensure rigorous and transparent reporting.^[11]^ Causal estimates were mainly derived using inverse-variance weighted (IVW),^[12]^ MR-Egger regression method (MR-Egger).^[13]^ Odds ratios (ORs) were calculated, with OR < 1 indicating protective effects and OR > 1 suggesting risk. Results were visualized via scatterplots (exposure-outcome SNP effects), forest plots (method-specific ORs), and funnel plots (symmetry assessment).

Sensitivity analyses included – heterogeneity: Cochran’s *Q* test^[14]^ (*P* < .05 indicates significant heterogeneity); horizontal pleiotropy: MR-Egger intercept test^[15]^ (*P* < .05 suggests bias); leave-one-out validation: Sequential SNP exclusion to identify influential variants.^[16]^

All analyses were performed in R (v4.2.1) using the TwoSampleMR package (v0.6.0).^[13]^ And the ggplot2 package was used for visualization.

## 3. Results

### 3.1. The causal impact of serum 25-hydroxyvitamin D levels on various oral diseases

Our primary analyses employing IVW and MR-Egger methods demonstrated consistent protective effects of serum 25-hydroxyvitamin D levels against specific oral diseases. For perioral dermatitis, multiple methods of MR approaches converged to show significant inverse associations: IVW (OR = 0.378), MR-Egger (OR = 0.179), weighted median (OR = 0.213), and maximum likelihood (OR = 0.376; *P* < .05).

In analyses of lip, oral cavity, pharyngeal malignancies, MR-Egger (OR = 0.197) and weighted mode (OR = 0.180) revealed statistically significant protective relationships (*P* < .05). Although the IVW estimate for malignancies showed borderline significance (OR = 0.399, *P* = .0885), the consistent directional effects across complementary methods strengthen the plausibility of this association.

**Figure 2.**
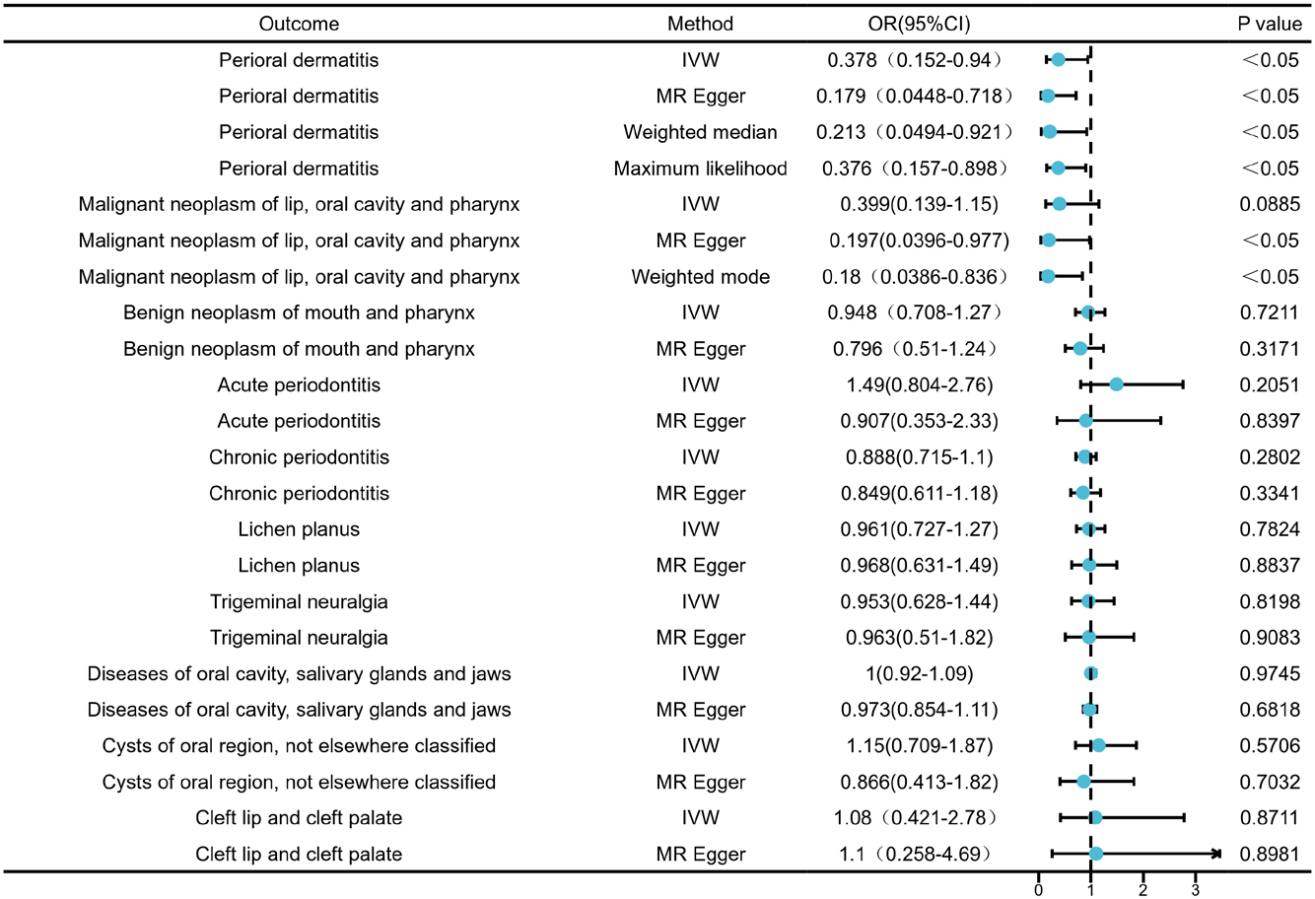
The forest plot of forward MR results (serum 25-hydroxyvitamin D levels were used as exposure). MR = Mendelian randomization.

Finally, no causal relationships were detected between serum 25-hydroxyvitamin D levels and other oral diseases, including benign neoplasm of mouth and pharynx, acute/chronic periodontitis, trigeminal neuralgia, lichen planus, diseases of oral cavity, salivary glands and jaws, cysts of oral region, not elsewhere classified and cleft lip and cleft palate. All MR estimates were comprehensively visualized through forest plots (Fig. 2).

**Figure 3.**
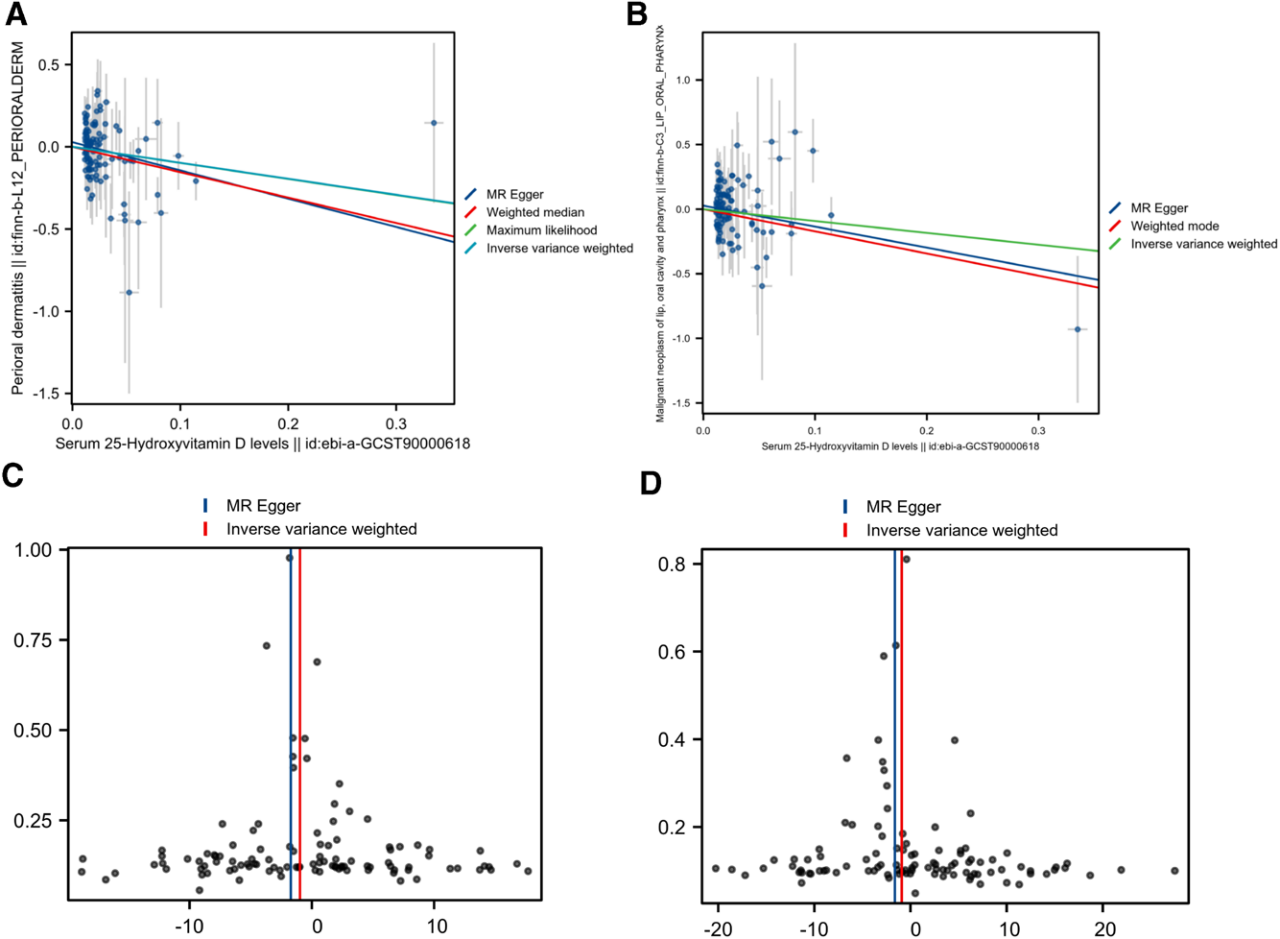
(A, B) Scatter plot of the MR analysis results. (C, D) Funnel plots of the MR analysis (A, C: perioral dermatitis were used as exposure the outcome; B, D: lip, oral cavity, and pharyngeal malignancies were used as exposure outcome). MR = Mendelian randomization.

Scatterplots of SNP effect sizes demonstrated inverse linear relationships between genetically predicted serum 25-hydroxyvitamin D levels and both perioral dermatitis (Fig. 3A) and oropharyngeal malignancies (Fig. 3B), visually confirming the protective genetic associations identified in MR analyses.

The symmetrical distribution patterns in funnel plots (Fig. 3C, D) provided empirical support for the validity of IVs, with balanced effect estimates across SNP magnitude strata. This distributional symmetry satisfies the key MR assumption of balanced pleiotropy, reinforcing the methodological robustness of our causal inferences.

### 3.2. Sensitivity tests have confirmed the robustness of the positive MR results

**Figure 4.**
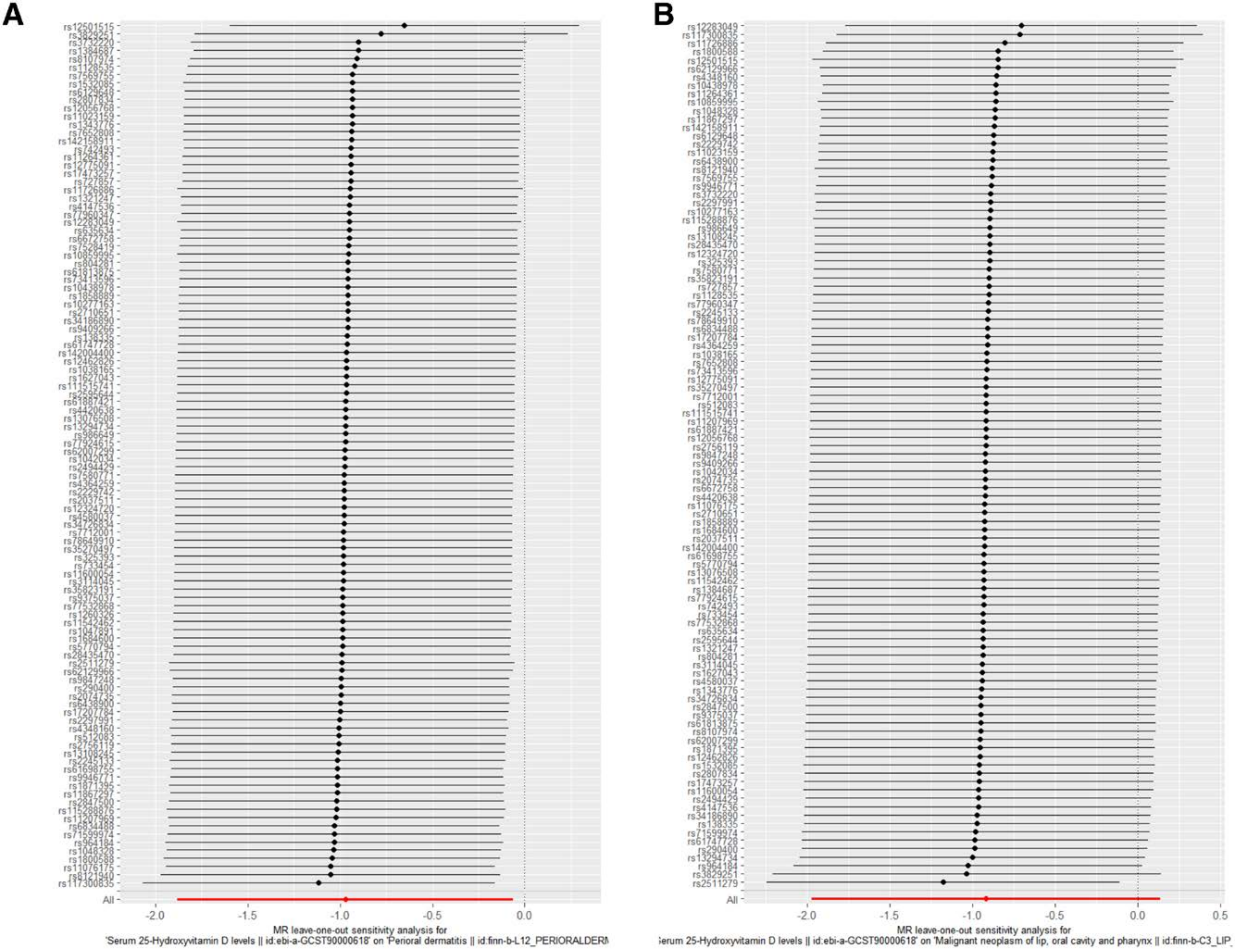
Forest plot of LOO analysis results. (A) Perioral dermatitis lip as outcome, (B) malignant neoplasm of lip, oral cavity and pharynx as outcome). LOO = leave-one-out.

Comprehensive sensitivity analyses were conducted to assess the robustness of MR estimates. Cochran’s *Q* statistics revealed no significant heterogeneity in the serum 25-hydroxyvitamin D levels-perioral dermatitis and Malignant neoplasm of lip, oral cavity and pharynx (Q_*P* value > 0.05; Table 2). Furthermore, MR-Egger intercept tests demonstrated no evidence of horizontal pleiotropy for these outcome pairs (*P* value > .05), supporting the exclusion restriction assumption that genetic instruments influence outcomes predominantly through serum 25-hydroxyvitamin D levels pathways (Table 3). Leave-one-out sensitivity analysis confirmed the absence of influential individual SNPs, with all iterative estimates remaining directionally consistent (Fig. 4).

**Table 2:** Check for heterogeneity.

Exposure	Outcome	Method	Q_*P* value
Serum 25-hydroxyvitamin D levels	Perioral dermatitis	IVW	0.22208748
Serum 25-hydroxyvitamin D levels	Perioral dermatitis	MR-Egger	0.243281238
Serum 25-hydroxyvitamin D levels	Malignant neoplasm of lip, oral cavity and pharynx	IVW	0.422885119
Serum 25-hydroxyvitamin D levels	Malignant neoplasm of lip, oral cavity and pharynx	MR-Egger	0.431897864

Q_*P* value > 0.05 indicates no heterogeneity.

IVW = inverse-variance weighted, MR = Mendelian randomization.

**Table 3:** Check for horizontal pleiotropy.

Exposure	Outcome	Method	Intercept	SE	*P* value
Serum serum 25-hydroxyvitamin D levels	Perioral dermatitis	MR-Egger	0.028126352	0.020202326	.16682163
Serum 25-hydroxyvitamin D levels	Malignant neoplasm of lip, oral cavity and pharynx	MR-Egger	0.027182178	0.023647768	.253080756

*P* value > .05 indicates no horizontal pleiotropy.

MR = Mendelian randomization, SE = standard error.

Sensitivity assessments for another oral diseases showed comparable null findings: neither heterogeneity (Q_*P* value > 0.05) nor horizontal pleiotropy (*P* > .05) were detected across any serum 25-hydroxyvitamin D levels-oral disease pairs (Tables S3 and S4, Supplemental Digital Content, https://links.lww.com/MD/Q701).

Collectively, these sensitivity analyses validate the robustness of our causal inferences, particularly for the observed protective relationships between serum 25-hydroxyvitamin D levels and both perioral dermatitis and lip, Malignant neoplasm of lip, oral cavity and pharynx ere used as exposure outcome.

### 3.3. MR analysis of various oral diseases as exposures and serum 25-hydroxyvitamin D levels as the outcome

**Figure 5.**
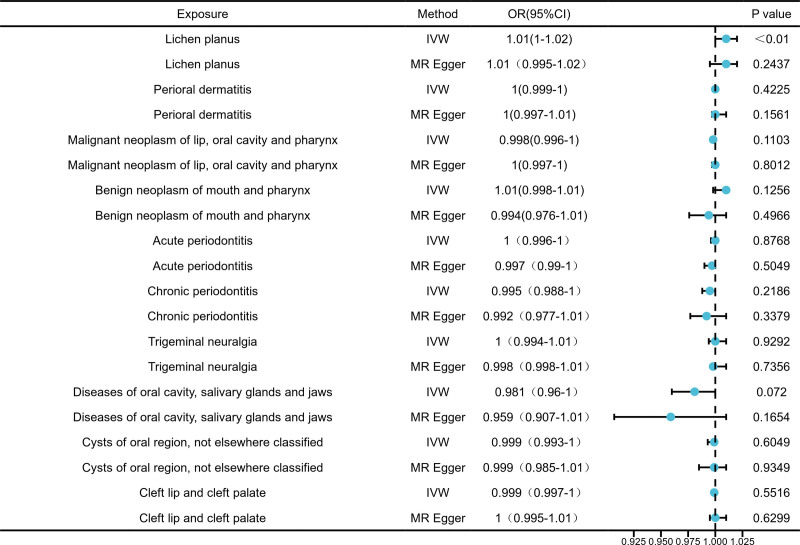
The forest plot of reverse MR results (serum 25-hydroxyvitamin D levels as outcome). MR = Mendelian randomization.

In bidirectional MR analyses evaluating oral disease-driven effects on serum 25-hydroxyvitamin D levels, only oral lichen planus (OLP) exhibited a marginally significant positive association with elevated serum 25-hydroxyvitamin D levels (OR = 1.010, 95% confidence interval [CI]: 1.000–1.020; Fig. 5).

Finally, we found no genetic evidence supporting reverse causal effects of perioral dermatitis, Malignant neoplasm of lip, oral cavity and pharynx, or other oral diseases on serum 25-hydroxyvitamin D levels (Fig. 5). The consistency of null findings was reinforced by: nonsignificant heterogeneity across all outcome-exposure pairs (Q_*P* value > 0.05; Table 4 and Table S5, Supplemental Digital Content, https://links.lww.com/MD/Q701). Absence of directional pleiotropy (*P* > .05; Table 5 and Table S6, Supplemental Digital Content, https://links.lww.com/MD/Q701).

**Table 4:** Check for heterogeneity.

Exposure	Outcome	Method	Q_*P* value
Lichen planus	Serum 25-hydroxyvitamin D levels	IVW	0.236732017
Lichen planus	Serum 25-hydroxyvitamin D levels	MR-Egger	0.192208275
Perioral dermatitis	Serum 25-hydroxyvitamin D levels	IVW	0.087696415
Perioral dermatitis	Serum 25-hydroxyvitamin D levels	MR-Egger	0.062294858
Malignant neoplasm of lip, oral cavity and pharynx	Serum 25-hydroxyvitamin D levels	IVW	0.164122027
Malignant neoplasm of lip, oral cavity and pharynx	Serum 25-hydroxyvitamin D levels	MR-Egger	0.267088854

Q_*P* value > 0.05 indicates no heterogeneity (serum 25-hydroxyvitamin D levels as outcome).

IVW = inverse-variance weighted, MR = Mendelian randomization.

**Table 5:** Check for horizontal pleiotropy.

Exposure	Outcome	Method	Intercept	SE	*P* value
Lichen planus	Serum 25-hydroxyvitamin D levels	MR-Egger	0.000443947	0.001477047	.767617555
Perioral dermatitis	Serum 25-hydroxyvitamin D levels	MR-Egger	−0.00035386	0.002047633	.865677906
Malignant neoplasm of lip, oral cavity and pharynx	Serum 25-hydroxyvitamin D levels	MR-Egger	−0.002902023	0.001871769	.159638049

*P* value > .05 indicates no horizontal pleiotropy. (Serum 25-hydroxyvitamin D levels as outcome).

MR = Mendelian randomization, SD = standard error.

## 4. Discussion

This bidirectional 2-sample MR study addresses 3 pivotal questions in the serum 25-hydroxyvitamin D levels-oral health nexus: whether genetically determined serum 25-hydroxyvitamin D levels exert causal effects on common oral pathologies; the potential reverse causation where oral diseases modulate vitamin D status; clinically informative nonlinear dose-response relationships that may guide supplementation thresholds. Leveraging large-scale genome-wide association study datasets, we employed rigorously selected genetic instruments and conducted comprehensive sensitivity analyses to ensure analytical robustness. Our findings elucidate the etiological mechanisms linking vitamin D metabolism and oral health disorders, while establishing methodological frameworks to resolve longstanding controversies about vitamin D’s therapeutic potential in dental practice. The identification of nonlinear biological effects further provides actionable insights for optimizing public health strategies and personalized supplementation protocols.

The forward-directional associations observed in perioral dermatitis align with vitamin D’s established immunomodulatory properties. 1,25-(OH)_2_D_3_ mediates immunosuppression through dual inhibition of T helper 1 activity: directly suppressing T cell production of interleukin-2 and interferon-gamma and blocking antigen-presenting cell-derived interleukin-12 secretion, synergistically disrupting T helper 1-driven immune activation while preserving immune homeostasis.^[17]^ For oropharyngeal malignancies, our findings corroborate epidemiological data suggesting vitamin D deficiency correlates with increased cancer risk, potentially through its role in regulating epithelial differentiation and apoptosis.^[6]^ Notably, the null associations observed in other oral diseases may reflect either true biological independence or insufficient statistical power for rare outcomes.

Our bidirectional MR analysis revealed a borderline association between OLP and elevated serum 25(OH)D levels (OR = 1.010, 95% CI: 1.000–1.020). Notably, the CI approximating the null value (1.000) and clinically negligible effect size strongly suggest stochastic variation rather than biological causation. This genetic evidence contrasts with observational reports linking vitamin D deficiency to OLP severity and pain intensity,^[18]^ as well as mechanistic studies demonstrating lipopolysaccharide-mediated downregulation of vitamin D receptor expression in oral keratinocytes – a recognized pathogenic pathway in OLP development.^[19]^ The discordance between MR findings and experimental data may reflect either false-positive associations in observational studies due to residual confounding (e.g., chronic inflammation affecting vitamin D metabolism) or limited statistical power in detecting nonlinear threshold effects through MR methodology.

Second, the absence of persistent reverse causation reinforces the robustness of our primary findings, effectively excluding the “sick-stopper” bias often confounding nutritional epidemiology.

Several limitations warrant consideration. First, although we excluded SNPs associated with classical confounders, residual pleiotropy through undisclosed pathways cannot be entirely excluded. This relates to the inherent challenge in fully verifying the core MR assumptions. Although the consistency of our results across multiple MR methods and the absence of significant horizontal pleiotropy in most analyses provide reassurance that such biases are unlikely to be substantial, the theoretical risks should be acknowledged. Second, our findings are based exclusively on European-ancestry genome-wide association study data, which has direct implications for their generalizability. The genetic architecture of vitamin D metabolism – including allele frequencies and effect sizes of instrumental variants – can differ substantially across diverse ethnic populations due to differences in genetics, lifestyle, and sun exposure. Consequently, the causal effects we identified may not be directly transferable to non-European populations. Applying these findings to other groups requires validation in multi-ethnic studies, as differential genetic effects could lead to either over- or under-estimation of the true causal relationship in those specific contexts. Third, the nonlinear relationship between vitamin D concentrations and oral health outcomes remains unexplored due to summary-level data constraints.

Our findings carry important clinical implications. The protective effects against oropharyngeal malignancies suggest vitamin D supplementation could serve as a cost-effective adjuvant in high-risk populations, though randomized controlled trials are needed to validate this hypothesis. For perioral dermatitis management, serum vitamin D monitoring may inform personalized prevention strategies.

Future research should prioritize: multi-ethnic replication studies using individual-level data; mechanistic investigations into tissue-specific vitamin D receptor signaling in oral epithelium; integration of proteomic data to delineate downstream effectors mediating these causal relationships.

## Author contributions

**Conceptualization:** Yuanxin Shi, Bin Chen, Guohui Bai.

**Data curation:** Yuanxin Shi, Guohui Bai.

**Funding acquisition:** Bin Chen, Guohui Bai.

**Investigation:** Yuanxin Shi, Xie Li.

**Methodology:** Yuanxin Shi, Yueyue Wang, Guohui Bai.

**Software:** Yuanxin Shi, Xie Li, Bin Chen, Yueyue Wang.

**Supervision:** Bin Chen, Guohui Bai.

**Visualization:** Yuanxin Shi, Xie Li.

**Writing – original draft:** Yuanxin Shi.

**Writing – review & editing:** Yuanxin Shi, Xie Li, Bin Chen, Yueyue Wang, Guohui Bai.

## Supplementary Material


