# Supplementary material for: Vitamin D and oral disease relationships: Insights from a bidirectional Mendelian randomization investigation
Source: Medicine (Baltimore). 2025 Nov 21;104(47):e46097. doi: 10.1097/MD.0000000000046097 (PMC12643689; doi:10.1097/MD.0000000000046097)
Supplement: Supplementary file 1 [file medi-104-e46097-s001.pdf]

Supplementary Table1. All instrumental variables used in the forward MR analysis

Exposure	SNP	eaf.exposure	F_statistic	mr_keep.exposure	pval.exposure
Serum 25-Hydroxyvitamin D levels || id:ebi-a-GCST90000618	rs11207969	0.351365	96.9429278475243	TRUE	7.1351e-23
Serum 25-Hydroxyvitamin D levels || id:ebi-a-GCST90000618	rs11264361	0.251408	55.8114269988048	TRUE	7.97444e-14
Serum 25-Hydroxyvitamin D levels || id:ebi-a-GCST90000618	rs61747728	0.0385611	33.0836637139684	TRUE	8.82755e-09
Serum 25-Hydroxyvitamin D levels || id:ebi-a-GCST90000618	rs2807834	0.68514	47.4442879816146	TRUE	5.65979e-12
Serum 25-Hydroxyvitamin D levels || id:ebi-a-GCST90000618	rs512083	0.462488	35.7656528837217	TRUE	2.22526e-09
Serum 25-Hydroxyvitamin D levels || id:ebi-a-GCST90000618	rs6672758	0.80023	40.4269845191042	TRUE	2.04094e-10
Serum 25-Hydroxyvitamin D levels || id:ebi-a-GCST90000618	rs2494429	0.822999	30.8395418552968	TRUE	2.80292e-08
Serum 25-Hydroxyvitamin D levels || id:ebi-a-GCST90000618	rs1343776	0.22133	54.4230460231719	TRUE	1.61659e-13
Serum 25-Hydroxyvitamin D levels || id:ebi-a-GCST90000618	rs7528419	0.22441	78.459386912677	TRUE	8.16582e-19
Serum 25-Hydroxyvitamin D levels || id:ebi-a-GCST90000618	rs115288876	0.0433211	250.187769049239	TRUE	2.3632e-56
Serum 25-Hydroxyvitamin D levels || id:ebi-a-GCST90000618	rs35823191	0.342049	118.095707050471	TRUE	1.65272e-27
Serum 25-Hydroxyvitamin D levels || id:ebi-a-GCST90000618	rs61813875	0.0248209	155.377006716814	TRUE	1.15824e-35
Serum 25-Hydroxyvitamin D levels || id:ebi-a-GCST90000618	rs1042034	0.792138	36.5994583300312	TRUE	1.45071e-09
Serum 25-Hydroxyvitamin D levels || id:ebi-a-GCST90000618	rs1260326	0.603925	90.3819996721775	TRUE	1.96336e-21
Serum 25-Hydroxyvitamin D levels || id:ebi-a-GCST90000618	rs35270497	0.176236	34.1593713919335	TRUE	5.07715e-09
Serum 25-Hydroxyvitamin D levels || id:ebi-a-GCST90000618	rs7569755	0.288618	36.5442128776769	TRUE	1.49242e-09
Serum 25-Hydroxyvitamin D levels || id:ebi-a-GCST90000618	rs2710651	0.526101	32.4406143363631	TRUE	1.2288e-08
Serum 25-Hydroxyvitamin D levels || id:ebi-a-GCST90000618	rs3732220	0.0852646	173.437790371865	TRUE	1.31341e-39
Serum 25-Hydroxyvitamin D levels || id:ebi-a-GCST90000618	rs727857	0.61181	32.9890364559504	TRUE	9.26659e-09
Serum 25-Hydroxyvitamin D levels || id:ebi-a-GCST90000618	rs7580771	0.175983	38.6182129811761	TRUE	5.15383e-10

Serum 25-Hydroxyvitamin D levels || id:ebi-a-GCST90000618	rs1047891	0.317078	37.7684484310038	TRUE	7.96453e-10
Serum 25-Hydroxyvitamin D levels || id:ebi-a-GCST90000618	rs7652808	0.649492	100.229655629069	TRUE	1.35738e-23
Serum 25-Hydroxyvitamin D levels || id:ebi-a-GCST90000618	rs9847248	0.712969	30.0595876663814	TRUE	4.19035e-08
Serum 25-Hydroxyvitamin D levels || id:ebi-a-GCST90000618	rs13076508	0.0535034	30.8564244216904	TRUE	2.77856e-08
Serum 25-Hydroxyvitamin D levels || id:ebi-a-GCST90000618	rs1128535	0.500049	65.4167133041377	TRUE	6.06457e-16
Serum 25-Hydroxyvitamin D levels || id:ebi-a-GCST90000618	rs6438900	0.256034	41.3471348461001	TRUE	1.27447e-10
Serum 25-Hydroxyvitamin D levels || id:ebi-a-GCST90000618	rs34186890	0.259637	45.7733469552619	TRUE	1.3277e-11
Serum 25-Hydroxyvitamin D levels || id:ebi-a-GCST90000618	rs6834488	0.422833	49.246639308312	TRUE	2.25684e-12
Serum 25-Hydroxyvitamin D levels || id:ebi-a-GCST90000618	rs13108245	0.386601	34.3379281102379	TRUE	4.63234e-09
Serum 25-Hydroxyvitamin D levels || id:ebi-a-GCST90000618	rs3114045	0.866137	55.3631113693274	TRUE	1.00207e-13
Serum 25-Hydroxyvitamin D levels || id:ebi-a-GCST90000618	rs78649910	0.105728	33.1955287875229	TRUE	8.33355e-09
Serum 25-Hydroxyvitamin D levels || id:ebi-a-GCST90000618	rs4348160	0.326968	142.765717921183	TRUE	6.61607e-33
Serum 25-Hydroxyvitamin D levels || id:ebi-a-GCST90000618	rs4147536	0.788613	35.3446191404298	TRUE	2.76179e-09
Serum 25-Hydroxyvitamin D levels || id:ebi-a-GCST90000618	rs4364259	0.198665	45.117424724024	TRUE	1.85524e-11
Serum 25-Hydroxyvitamin D levels || id:ebi-a-GCST90000618	rs71599974	0.148048	80.883179455127	TRUE	2.39497e-19
Serum 25-Hydroxyvitamin D levels || id:ebi-a-GCST90000618	rs12501515	0.58966	1455.88129777357	TRUE	1e-200
Serum 25-Hydroxyvitamin D levels || id:ebi-a-GCST90000618	rs11726886	0.290783	566.601420385195	TRUE	3.08319e-125
Serum 25-Hydroxyvitamin D levels || id:ebi-a-GCST90000618	rs7712001	0.440176	33.5193354189205	TRUE	7.05472e-09
Serum 25-Hydroxyvitamin D levels || id:ebi-a-GCST90000618	rs986649	0.321628	34.8796556288667	TRUE	3.50728e-09
Serum 25-Hydroxyvitamin D levels || id:ebi-a-GCST90000618	rs17207784	0.324195	38.6220489580403	TRUE	5.14375e-10
Serum 25-Hydroxyvitamin D levels || id:ebi-a-GCST90000618	rs1321247	0.101578	43.4445866478114	TRUE	4.36214e-11
Serum 25-Hydroxyvitamin D levels || id:ebi-a-GCST90000618	rs12153819	0.123154	33.2370567828268	TRUE	8.15831e-09

Serum 25-Hydroxyvitamin D levels || id:ebi-a-GCST90000618	rs742493	0.112871	32.7686585580458	TRUE	1.03805e-08
Serum 25-Hydroxyvitamin D levels || id:ebi-a-GCST90000618	rs2245133	0.164339	60.3841171524298	TRUE	7.80369e-15
Serum 25-Hydroxyvitamin D levels || id:ebi-a-GCST90000618	rs9375037	0.443168	32.4721829197148	TRUE	1.20895e-08
Serum 25-Hydroxyvitamin D levels || id:ebi-a-GCST90000618	rs1858889	0.502532	43.8798186268583	TRUE	3.4914e-11
Serum 25-Hydroxyvitamin D levels || id:ebi-a-GCST90000618	rs2595644	0.384889	34.1997800436872	TRUE	4.9737e-09
Serum 25-Hydroxyvitamin D levels || id:ebi-a-GCST90000618	rs7784802	0.358913	39.6381447547776	TRUE	3.05675e-10
Serum 25-Hydroxyvitamin D levels || id:ebi-a-GCST90000618	rs10277163	0.254715	37.1805788387753	TRUE	1.07676e-09
Serum 25-Hydroxyvitamin D levels || id:ebi-a-GCST90000618	rs804281	0.583498	59.5386897748306	TRUE	1.19922e-14
Serum 25-Hydroxyvitamin D levels || id:ebi-a-GCST90000618	rs1384687	0.132271	31.6739745897938	TRUE	1.82331e-08
Serum 25-Hydroxyvitamin D levels || id:ebi-a-GCST90000618	rs12056768	0.584044	126.294753468185	TRUE	2.65094e-29
Serum 25-Hydroxyvitamin D levels || id:ebi-a-GCST90000618	rs34726834	0.252209	35.6001253875498	TRUE	2.42237e-09
Serum 25-Hydroxyvitamin D levels || id:ebi-a-GCST90000618	rs9409266	0.862222	32.419268284107	TRUE	1.2426e-08
Serum 25-Hydroxyvitamin D levels || id:ebi-a-GCST90000618	rs13294734	0.466128	37.2808200640521	TRUE	1.02277e-09
Serum 25-Hydroxyvitamin D levels || id:ebi-a-GCST90000618	rs635634	0.186573	33.3876465386849	TRUE	7.55092e-09
Serum 25-Hydroxyvitamin D levels || id:ebi-a-GCST90000618	rs2398113	0.423526	32.6573453567658	TRUE	1.09921e-08
Serum 25-Hydroxyvitamin D levels || id:ebi-a-GCST90000618	rs12775091	0.213441	39.4722781431235	TRUE	3.32767e-10
Serum 25-Hydroxyvitamin D levels || id:ebi-a-GCST90000618	rs2297991	0.718478	31.9703221596674	TRUE	1.5656e-08
Serum 25-Hydroxyvitamin D levels || id:ebi-a-GCST90000618	rs77532868	0.0521528	32.3565829056759	TRUE	1.28316e-08
Serum 25-Hydroxyvitamin D levels || id:ebi-a-GCST90000618	rs144965707	0.0618296	68.1391817955032	TRUE	1.52335e-16
Serum 25-Hydroxyvitamin D levels || id:ebi-a-GCST90000618	rs1627043	0.0332414	73.8354335712145	TRUE	8.4918e-18
Serum 25-Hydroxyvitamin D levels || id:ebi-a-GCST90000618	rs2847500	0.123204	53.3631279490397	TRUE	2.77268e-13
Serum 25-Hydroxyvitamin D levels || id:ebi-a-GCST90000618	rs17473257	0.0172466	61.4289211675291	TRUE	4.59092e-15

Serum 25-Hydroxyvitamin D levels || id:ebi-a-GCST90000618	rs117300835	0.0133061	1429.48096170292	TRUE	1e-200
Serum 25-Hydroxyvitamin D levels || id:ebi-a-GCST90000618	rs2511279	0.960373	355.296434632629	TRUE	2.97715e-79
Serum 25-Hydroxyvitamin D levels || id:ebi-a-GCST90000618	rs3829251	0.133277	1474.54701347573	TRUE	1e-200
Serum 25-Hydroxyvitamin D levels || id:ebi-a-GCST90000618	rs11023159	0.0325143	70.9158460743887	TRUE	3.72735e-17
Serum 25-Hydroxyvitamin D levels || id:ebi-a-GCST90000618	rs733454	0.0991663	30.7517369488592	TRUE	2.93265e-08
Serum 25-Hydroxyvitamin D levels || id:ebi-a-GCST90000618	rs111515741	0.0173081	39.1365841838515	TRUE	3.95185e-10
Serum 25-Hydroxyvitamin D levels || id:ebi-a-GCST90000618	rs12283049	0.234433	550.538447803311	TRUE	9.61612e-122
Serum 25-Hydroxyvitamin D levels || id:ebi-a-GCST90000618	rs11600054	0.0100965	45.1338802760091	TRUE	1.83992e-11
Serum 25-Hydroxyvitamin D levels || id:ebi-a-GCST90000618	rs964184	0.867234	185.225820812174	TRUE	3.50429e-42
Serum 25-Hydroxyvitamin D levels || id:ebi-a-GCST90000618	rs61887421	0.0300871	37.7487241591969	TRUE	8.04674e-10
Serum 25-Hydroxyvitamin D levels || id:ebi-a-GCST90000618	rs7955128	0.52053	41.053710236305	TRUE	1.48095e-10
Serum 25-Hydroxyvitamin D levels || id:ebi-a-GCST90000618	rs1038165	0.579451	31.3582352931985	TRUE	2.14541e-08
Serum 25-Hydroxyvitamin D levels || id:ebi-a-GCST90000618	rs73413596	0.0740317	33.0135527681525	TRUE	9.15103e-09
Serum 25-Hydroxyvitamin D levels || id:ebi-a-GCST90000618	rs28435470	0.663204	30.526588142573	TRUE	3.29367e-08
Serum 25-Hydroxyvitamin D levels || id:ebi-a-GCST90000618	rs57601828	0.394508	30.7350600099305	TRUE	2.95801e-08
Serum 25-Hydroxyvitamin D levels || id:ebi-a-GCST90000618	rs1871395	0.152679	51.9403583350133	TRUE	5.72137e-13
Serum 25-Hydroxyvitamin D levels || id:ebi-a-GCST90000618	rs2171427	0.156491	34.5022492844911	TRUE	4.25706e-09
Serum 25-Hydroxyvitamin D levels || id:ebi-a-GCST90000618	rs10859995	0.579841	450.897686854755	TRUE	4.60045e-100
Serum 25-Hydroxyvitamin D levels || id:ebi-a-GCST90000618	rs4580037	0.285578	36.3096746144943	TRUE	1.68306e-09
Serum 25-Hydroxyvitamin D levels || id:ebi-a-GCST90000618	rs2756119	0.38143	33.1093797599929	TRUE	8.71224e-09
Serum 25-Hydroxyvitamin D levels || id:ebi-a-GCST90000618	rs142004400	0.0342066	30.6989676903643	TRUE	3.01328e-08
Serum 25-Hydroxyvitamin D levels || id:ebi-a-GCST90000618	rs1532085	0.616672	146.818348116576	TRUE	8.59805e-34

Serum 25-Hydroxyvitamin D levels || id:ebi-a-GCST90000618	rs1800588	0.214964	152.582502417344	TRUE	4.72607e-35
Serum 25-Hydroxyvitamin D levels || id:ebi-a-GCST90000618	rs62007299	0.712958	30.6324783536729	TRUE	3.11853e-08
Serum 25-Hydroxyvitamin D levels || id:ebi-a-GCST90000618	rs325393	0.278218	35.9427835175896	TRUE	2.03226e-09
Serum 25-Hydroxyvitamin D levels || id:ebi-a-GCST90000618	rs12324720	0.174629	31.1035868138508	TRUE	2.44625e-08
Serum 25-Hydroxyvitamin D levels || id:ebi-a-GCST90000618	rs1684600	0.298695	31.9376716568481	TRUE	1.59188e-08
Serum 25-Hydroxyvitamin D levels || id:ebi-a-GCST90000618	rs11542462	0.133511	69.025336380881	TRUE	9.72076e-17
Serum 25-Hydroxyvitamin D levels || id:ebi-a-GCST90000618	rs77924615	0.194374	34.6524574842936	TRUE	3.94176e-09
Serum 25-Hydroxyvitamin D levels || id:ebi-a-GCST90000618	rs11076175	0.175666	73.5839945825733	TRUE	9.64273e-18
Serum 25-Hydroxyvitamin D levels || id:ebi-a-GCST90000618	rs11867297	0.38533	41.8085655065681	TRUE	1.00649e-10
Serum 25-Hydroxyvitamin D levels || id:ebi-a-GCST90000618	rs61698755	0.560041	31.2628502893978	TRUE	2.25372e-08
Serum 25-Hydroxyvitamin D levels || id:ebi-a-GCST90000618	rs9946771	0.0663426	32.9470452293042	TRUE	9.47022e-09
Serum 25-Hydroxyvitamin D levels || id:ebi-a-GCST90000618	rs2037511	0.165924	41.9398852102232	TRUE	9.41239e-11
Serum 25-Hydroxyvitamin D levels || id:ebi-a-GCST90000618	rs77960347	0.0127013	33.6693865993472	TRUE	6.53221e-09
Serum 25-Hydroxyvitamin D levels || id:ebi-a-GCST90000618	rs10438978	0.820277	42.4266478378955	TRUE	7.33838e-11
Serum 25-Hydroxyvitamin D levels || id:ebi-a-GCST90000618	rs1048328	0.0797967	70.1214323743884	TRUE	5.57571e-17
Serum 25-Hydroxyvitamin D levels || id:ebi-a-GCST90000618	rs142158911	0.111826	66.0357854294674	TRUE	4.42792e-16
Serum 25-Hydroxyvitamin D levels || id:ebi-a-GCST90000618	rs12462826	0.369566	39.025716143872	TRUE	4.18312e-10
Serum 25-Hydroxyvitamin D levels || id:ebi-a-GCST90000618	rs4420638	0.176831	52.6671829527546	TRUE	3.95094e-13
Serum 25-Hydroxyvitamin D levels || id:ebi-a-GCST90000618	rs8107974	0.0763013	86.5474759346317	TRUE	1.36427e-20
Serum 25-Hydroxyvitamin D levels || id:ebi-a-GCST90000618	rs62129966	0.160854	489.773015165402	TRUE	1.59588e-108
Serum 25-Hydroxyvitamin D levels || id:ebi-a-GCST90000618	rs1841850	0.117085	92.5022506765039	TRUE	6.72667e-22
Serum 25-Hydroxyvitamin D levels || id:ebi-a-GCST90000618	rs8121940	0.19762	292.056458174531	TRUE	1.77052e-65

Serum 25-Hydroxyvitamin D levels || id:ebi-a-GCST90000618	rs6129648	0.379837	44.5789589266524	TRUE	2.44287e-11
Serum 25-Hydroxyvitamin D levels || id:ebi-a-GCST90000618	rs290400	0.665472	36.6486632116471	TRUE	1.41465e-09
Serum 25-Hydroxyvitamin D levels || id:ebi-a-GCST90000618	rs2229742	0.104659	56.8309500259647	TRUE	4.75007e-14
Serum 25-Hydroxyvitamin D levels || id:ebi-a-GCST90000618	rs138335	0.658587	40.9536929188226	TRUE	1.55887e-10
Serum 25-Hydroxyvitamin D levels || id:ebi-a-GCST90000618	rs2074735	0.0648365	50.4606777788905	TRUE	1.21563e-12
Serum 25-Hydroxyvitamin D levels || id:ebi-a-GCST90000618	rs5770794	0.3143	36.2474312920299	TRUE	1.738e-09

Supplementary Table2. 4 SNPs that were excluded （Malignant neoplasm of lip, oral cavity and pharynx are used as exposure outcome）

SNP	Exposure	Outcome	Confounder
rs7528419	Serum 25-Hydroxyvitamin D levels	Malignant neoplasm of lip, oral cavity and pharynx	LDL cholesterol levels in current drinkers
rs1260326	Serum 25-Hydroxyvitamin D levels	Malignant neoplasm of lip, oral cavity and pharynx	Alcohol consumption (drinks per week)
rs1047891	Serum 25-Hydroxyvitamin D levels	Malignant neoplasm of lip, oral cavity and pharynx	HDL cholesterol levels in current drinkers
rs10859995	Serum 25-Hydroxyvitamin D levels	Malignant neoplasm of lip, oral cavity and pharynx	Vitamin D insufficiency

Supplementary Table 3. Check for heterogeneity. Q_pval > 0.05 indicates no heterogeneity.（Forward Mendelian randomization）

Exposure	Outcome	Method	Q	Q_df	Q_pval
Serum 25-Hydroxyvitamin D levels	Perioral dermatitis	IVW	115.7747556	105	0.22208748
Serum 25-Hydroxyvitamin D levels	Perioral dermatitis	MR Egger	113.6564683	104	0.243281238
Serum 25-Hydroxyvitamin D levels	Malignant neoplasm of lip, oral cavity and pharynx	IVW	104.1270523	102	0.422885119
Serum 25-Hydroxyvitamin D levels	Malignant neoplasm of lip, oral cavity and pharynx	MR Egger	102.7824746	101	0.431897864
Serum 25-Hydroxyvitamin D levels	Benign neoplasm of mouth and pharynx	IVW	99.93889885	102	0.539237447
Serum 25-Hydroxyvitamin D levels	Benign neoplasm of mouth and pharynx	MR Egger	98.89213327	101	0.540757741
Serum 25-Hydroxyvitamin D levels	Acute periodontitis	IVW	85.3643904	105	0.919732624
Serum 25-Hydroxyvitamin D levels	Acute periodontitis	MR Egger	83.51200963	104	0.930465501
Serum 25-Hydroxyvitamin D levels	Chronic periodontitis	IVW	90.18612365	105	0.848055813
Serum 25-Hydroxyvitamin D levels	Chronic periodontitis	MR Egger	90.06466937	104	0.833062294
Serum 25-Hydroxyvitamin D levels	Lichen planus	IVW	103.8533277	105	0.513302499
Serum 25-Hydroxyvitamin D levels	Lichen planus	MR Egger	103.8513299	104	0.485665743
Serum 25-Hydroxyvitamin D levels	Trigeminal neuralgia	IVW	81.52908006	105	0.956558176
Serum 25-Hydroxyvitamin D levels	Trigeminal neuralgia	MR Egger	81.52708281	104	0.949498391
Serum 25-Hydroxyvitamin D levels	Diseases of oral cavity, salivary glands and jaws	IVW	110.1223893	105	0.346890206
Serum 25-Hydroxyvitamin D levels	Diseases of oral cavity, salivary glands and jaws	MR Egger	109.7781933	104	0.330151983
Serum 25-Hydroxyvitamin D levels	Cysts of oral region, not elsewhere classified	IVW	107.5943186	105	0.411572056
Serum 25-Hydroxyvitamin D levels	Cysts of oral region, not elsewhere classified	MR Egger	106.5756609	104	0.41165556
Serum 25-Hydroxyvitamin D levels	Cleft lip and cleft palate	IVW	121.6897281	105	0.126888227
Serum 25-Hydroxyvitamin D	Cleft lip and cleft palate	MR Egger	121.6886572	104	0.113378

levels

Supplementary Table 4. Check for Horizontal pleiotropy. Pval > 0.05 indicates no horizontal pleiotropy. （Forward Mendelian randomization）

Exposure	Outcome	Method	Intercept	Se	Pval
Serum 25-Hydroxyvitamin D levels	Perioral dermatitis	MR Egger	0.028126352	0.020202326	0.16682163
Serum 25-Hydroxyvitamin D levels	Malignant neoplasm of lip, oral cavity and pharynx	MR Egger	0.027182178	0.023647768	0.253080756
Serum 25-Hydroxyvitamin D levels	Benign neoplasm of mouth and pharynx	MR Egger	0.006708163	0.006556603	0.308697484
Serum 25-Hydroxyvitamin D levels	Acute periodontitis	MR Egger	0.018741367	0.01377007	0.176448638
Serum 25-Hydroxyvitamin D levels	Chronic periodontitis	MR Egger	0.00167986	0.004820217	0.728167017
Serum 25-Hydroxyvitamin D levels	Lichen planus	MR Egger	-0.000278847	0.006238638	0.964434683
Serum 25-Hydroxyvitamin D levels	Trigeminal neuralgia	MR Egger	-0.000414975	0.009285507	0.964439647
Serum 25-Hydroxyvitamin D levels	Diseases of oral cavity, salivary glands and jaws	MR Egger	0.001085205	0.001900422	0.569208882
Serum 25-Hydroxyvitamin D levels	Cysts of oral region, not elsewhere classified	MR Egger	0.010785511	0.010817799	0.321071221
Serum 25-Hydroxyvitamin D levels	Cleft lip and cleft palate	MR Egger	-0.000639499	0.021138564	0.975923497
Serum 25-Hydroxyvitamin D levels	Mouth/teeth dental problems: Mouth ulcers	MR Egger	0.000102491	0.000138526	0.461029695

Supplementary Table 5. Check for heterogeneity. Q_pval > 0.05 indicates no heterogeneity.（Reverse Mendelian randomization）

Exposure	Outcome	Method	Q	Q_df	Q_pval
Lichen planus	Serum 25-Hydroxyvitamin D levels	IVW	20.77033721	17	0.236732017
Lichen planus	Serum 25-Hydroxyvitamin D levels	MR Egger	20.65372304	16	0.192208275
Perioral dermatitis	Serum 25-Hydroxyvitamin D levels	IVW	20.31211223	13	0.087696415
Perioral dermatitis	Serum 25-Hydroxyvitamin D levels	MR Egger	20.26168628	12	0.062294858
Malignant neoplasm of lip, oral cavity and pharynx	Serum 25-Hydroxyvitamin D levels	IVW	12.96663032	9	0.164122027
Malignant neoplasm of lip, oral cavity and pharynx	Serum 25-Hydroxyvitamin D levels	MR Egger	9.970693877	8	0.267088854
Benign neoplasm of mouth and pharynx	Serum 25-Hydroxyvitamin D levels	IVW	11.27348697	10	0.336611646
Benign neoplasm of mouth and pharynx	Serum 25-Hydroxyvitamin D levels	MR Egger	8.990044722	9	0.438193659
Acute periodontitis	Serum 25-Hydroxyvitamin D levels	IVW	3.871761565	9	0.919648178
Acute periodontitis	Serum 25-Hydroxyvitamin D levels	MR Egger	3.378824447	8	0.908387939
Chronic periodontitis	Serum 25-Hydroxyvitamin D levels	IVW	19.10203361	18	0.385565716
Chronic periodontitis	Serum 25-Hydroxyvitamin D levels	MR Egger	18.86220529	17	0.336498574
Trigeminal neuralgia	Serum 25-Hydroxyvitamin D levels	IVW	14.05354841	8	0.08038033
Trigeminal neuralgia	Serum 25-Hydroxyvitamin D levels	MR Egger	13.76821612	7	0.055460805
Diseases of oral cavity, salivary glands and jaws	Serum 25-Hydroxyvitamin D levels	IVW	8.725119612	14	0.848246061
Diseases of oral cavity, salivary glands and jaws	Serum 25-Hydroxyvitamin D levels	MR Egger	8.006041108	13	0.843206485
Cysts of oral region, not elsewhere classified	Serum 25-Hydroxyvitamin D levels	IVW	25.75799831	12	0.011612372
Cysts of oral region, not elsewhere classified	Serum 25-Hydroxyvitamin D levels	MR Egger	25.72533676	11	0.007128824
Cleft lip and cleft palate	Serum 25-Hydroxyvitamin D levels	IVW	17.97359642	12	0.116494579
Cleft lip and cleft palate	Serum 25-Hydroxyvitamin D levels	MR Egger	17.01797333	11	0.107345062

Supplementary Table 6. Check for Horizontal pleiotropy. Pval > 0.05 indicates no horizontal pleiotropy.（Reverse Mendelian randomization）

Exposure	Outcome	Method	Intercept	Se	Pval
Lichen planus	Serum 25-Hydroxyvitamin D levels	MR Egger	0.000443947	0.001477047	0.767617555
Perioral dermatitis	Serum 25-Hydroxyvitamin D levels	MR Egger	-0.00035386	0.002047633	0.865677906
Malignant neoplasm of lip, oral cavity and pharynx	Serum 25-Hydroxyvitamin D levels	MR Egger	-0.002902023	0.001871769	0.159638049
Benign neoplasm of mouth and pharynx	Serum 25-Hydroxyvitamin D levels	MR Egger	0.003202577	0.002119359	0.165047135
Acute periodontitis	Serum 25-Hydroxyvitamin D levels	MR Egger	0.001299989	0.001851586	0.502532171
Chronic periodontitis	Serum 25-Hydroxyvitamin D levels	MR Egger	0.000673775	0.001449227	0.647888113
Trigeminal neuralgia	Serum 25-Hydroxyvitamin D levels	MR Egger	0.000986516	0.002590112	0.714589574
Diseases of oral cavity, salivary glands and jaws	Serum 25-Hydroxyvitamin D levels	MR Egger	0.001814998	0.002140366	0.411787803
Cysts of oral region, not elsewhere classified	Serum 25-Hydroxyvitamin D levels	MR Egger	-0.00034906	0.002953699	0.908057533
Cleft lip and cleft palate	Serum 25-Hydroxyvitamin D levels	MR Egger	-0.00270629	0.003443408	0.448505628
